# Family planning as a critical component of sustainable global development

**DOI:** 10.3402/gha.v8.29978

**Published:** 2015-11-09

**Authors:** Babatunde Osotimehin

**Affiliations:** United Nations Population Fund, Under-Secretary-General of the United Nations

In September 2015, under the leadership of United Nations Secretary-General Ban Ki-Moon, the international community took a huge step forward for people, the planet, and prosperity with the adoption of the 2030 Agenda for Sustainable Development. The 17 Sustainable Development Goals (SDGs) pave a brave path towards a world of equity and inclusion, health, including sexual and reproductive health and reproductive rights, education, and greater equality (1).

Over the 1990–2015 timeframe of the Millennium Development Goals and, particularly since the 1994 International Conference on Population and Development, considerable progress has been made in women's sexual and reproductive health, including increases in contraceptive use globally, expanded access to skilled maternity care, and the reduction of new HIV infections and maternal and newborn deaths. The secretary-general's ‘Every Woman Every Child’ strategy (2) has catalysed increased leadership and commitment from governments and strong support from all partners, including United Nations agencies, non-governmental organisations, foundations, academia and professional associations.

Behind the positive global trends, however, lie significant differences among and within countries. For example, around 225 million women in low- and middle-income countries (LMICs) who do not want to become pregnant are not using modern contraception. It is estimated that 30 million unplanned births and 40 million abortions, half of them illegal and unsafe, occur annually. An estimated 499 million new sexually transmitted infections (excluding HIV) occur annually, approximately half among girls and women (2, 3).

The papers in this Special Issue reinforce the centrality of universal access to modern contraception within the SDGs and targets set for 2030 (1). Indeed the research and findings presented here point to an ecological correlation between satisfying demand for family planning using modern contraceptives and economic development (4). To reach the proposed benchmark of 75%, demand satisfied with modern methods of contraception would need to increase by 2.2 percentage points annually between 2014 and 2030 – more than double the current projections on average across the 63 countries analysed. Such rapid progress would require significant effort, particularly to meet needs among adolescent girls. If the 75% benchmark were achieved, 334 million women across the LMICs studied would use a modern contraceptive method by 2030, compared to 226 million women in 2014.

Strategies to increase family planning coverage (FPC) have to be backed up by effective metrics for assessing progress. For this purpose, a new FPC indicator has been developed by the authors in this special issue, based on the prevalence of contraceptive use (5).

Although coverage is an important indicator, meeting unmet needs and ensuring universal access to human rights-based family planning will not be achieved without addressing equity and quality issues. In this Special Issue, some authors have taken a detailed look at differences in access and use at the subnational level and over time in three countries: Burkina Faso, Ethiopia, and Nigeria (6–8). In all three cases they discovered substantial variations in modern contraceptive use between rural and urban areas and by other socio-economic factors. Consistently across all three countries the results confirm an association between fertility history and modern contraceptive use, as well as between low modern contraceptive use and higher birth risks, leading to increased child mortality. Moreover, women living in rural areas have significantly higher odds of avoidable birth risks (and hence child mortality) compared to their counterparts living in urban areas. In Burkina Faso short birth spacing ranked as the highest risk in relation to child deaths (6).

As the world aspires to a situation in which every adolescent girl and woman has easy access to comprehensive sexuality education and contraceptive services, studies here on contraceptive use among sexually active adolescents in Burkina Faso, Ethiopia, and Nigeria (9) and postpartum family planning uptake in Ethiopia, Malawi, and Nigeria (10) also highlighted equity and quality issues. Health systems must address these issues as the global community moves towards universal access. Marriage at very young ages is not only a human rights issue in itself, but also a barrier to modern contraceptive use, and it thus disempowers girls. Adolescent girls experience significant inequality in access to modern contraception by education, residence, and wealth quintile. The results from Ethiopia, however, show that leadership and commitment at the country level can bring change. The authors reported a significant and systematic reduction of inequalities, but also a narrowing of the equity gap, most notably for childbearing adolescents with no education or living in rural areas.

Mortality risks associated with low birth spacing have been further studied to assess potential confounding in the association between short-birth intervals and increased neonatal, infant, and child mortality in order to better inform attributable effects (11). After adjusting for confounding, the authors reported that neonatal, infant, and child mortality remained strongly and significantly related to short birth intervals, albeit with a one third reduction in the attributable risk ratio.

Any resilient health system must take into account the provision of reproductive, maternal, newborn, and adolescent health services across the life cycle, including easy access to family planning. The findings in these papers point to missed opportunities for integrating maternal and newborn health, including failure to combine child immunisation with family planning, as the factor most associated with the non-uptake of modern contraception in the postpartum period (10).

As we celebrate the historic adoption of the SDGs and look ahead to their implementation, access to family planning represents an important entry point and a marker for universal access to reproductive health and rights. Access to modern contraception reduces the risks of maternal and newborn deaths (11) as well as reducing lifetime parity, and thus it affects health, life expectancy, and the dependency ratio. The availability of family planning affects education prospects and human capital among adolescent girls by preventing teenage pregnancies and enabling girls to stay in school. It could also improve access to food and reduce hunger by reducing the dependency ratio. Urbanisation and population dynamics are intrinsically linked, particularly internal and external migration, which have poverty as a root cause. Access to modern contraception can spur the economy, protect the environment, and contribute to overall poverty reduction.

The research findings in this Special Issue, which come from distinguished scientists in United Nations agencies, non-governmental organisations, and universities, present a range of global and local policy and programmatic priorities to address measurement, coverage, impact, quality and equity issues in family planning services, which are integral to the global sustainable development agenda. This is particularly important for countries in sub-Saharan Africa. We thus call on world leaders and financiers to join forces and work towards the future we want, a future in which every pregnancy is wanted, every birth is safe, and every young person's potential is fulfilled.

*Babatunde Osotimehin* Executive Director United Nations Population Fund Under-Secretary-General of the United Nations
